# Catalytic Behavior of Lipase Immobilized onto Congo Red and PEG-Decorated Particles

**DOI:** 10.3390/molecules19068610

**Published:** 2014-06-24

**Authors:** Rubens A. Silva, Ana M. Carmona-Ribeiro, Denise F. S. Petri

**Affiliations:** Instituto de Química, Universidade de São Paulo, Av. Prof. Lineu Prestes 748, São Paulo 05508-000, SP, Brazil; E-Mails: rubens.silva@usp.br (R.A.S.); amcr@usp.br (A.M.C.-R.)

**Keywords:** lipase, poly(ethylene glycol), Congo red, kinetic parameters, bioconjugation

## Abstract

Poly(ethylene glycol) (PEG)-decorated polystyrene (PS) nanoparticles with mean hydrodynamic diameter (*D*) and zeta–potential (*ζ*) of (286 ± 15) nm and (−50 ± 5) mV, respectively, were modified by the adsorption of Congo red (CR). The PS/PEG/CR particles presented *D* and *ζ* values of (290 ± 19) nm and (−36 ± 5) mV, respectively. The adsorption of lipase onto PS/PEG or PS/PEG/CR particles at (24 ± 1) °C and pH 7 changed the mean *D* value to (380 ± 20) and (405 ± 11) nm, respectively, and *ζ* value to (−32 ± 4) mV and (−25 ± 2) mV, respectively. The kinetic parameters of the hydrolysis of *p*-nitrophenyl butyrate were determined for free lipase, lipase immobilized onto PS/PEG and PS/PEG/CR particles. Lipase on PS/PEG/CR presented the largest Michaelis-Menten constant (*K_M_*), but also the highest V_max_ and *k*_cat_ values. Moreover, it could be recycled seven times, losing a maximum 10% or 30% of the original enzymatic activity at 40 °C or 25 °C, respectively. Although lipases immobilized onto PS/PEG particles presented the smallest *K_M_* values, the reactions were comparatively the slowest and recycling was not possible. Hydrolysis reactions performed in the temperature range of 25 °C to 60 °C with free lipases and lipases immobilized onto PS/PEG/CR particles presented an optimal temperature at 40 °C. At 60 °C free lipases and lipases immobilized onto PS/PEG/CR presented ~80% and ~50% of the activity measured at 40 °C, indicating good thermal stability. Bioconjugation effects between CR and lipase were evidenced by circular dichroism spectroscopy and spectrophotometry. CR molecules mediate the open state conformation of the lipase lid and favor the substrate approaching.

## 1. Introduction

Immobilized enzymes are “green” catalysts for many reactions of industrial interest. They can be advantageous compared to free enzymes because: (i) immobilization improves their stability; (ii) subsequent reuse is possible; (iii) inhibition by products or sub-products is avoided; (iv) optimum pHs and temperatures might shift to more convenient values. However, the immobilized enzymes must maintain their active sites in a suitable orientation in order to retain their catalytic properties and the costs related to the support and to the immobilization process should be as low as possible [1].

Lipases are probably the most used enzymes in hydrolysis, esterification, acidolysis and alcoholysis reactions. Some of the reasons for this are their low cost, compared to other enzymes, high stability in organic media or emulsions or under extreme reactions conditions (pH, ionic strength and temperature), broad substrate specificity and the catalysis of regio- and enantioselective reactions [2]. Lipases are hydrolases composed of a core of predominantly parallel β strands surrounded by α helices [3]. The active site of lipases is formed by a catalytic triad consisting of the amino acids serine, histidine and aspartic acid/glutamic acid [4], which is covered by a lid or flap composed of an amphiphilic α- helix peptide sequence. In the presence of hydrophobic substrates, the lid is displaced and the active site is exposed. Lipases have been successfully immobilized in a large variety of surfaces by different techniques. The literature is plenty of excellent reviews on this subject, as for instance, references [1,5,6,7,8,9]. In a general form, enzymes can be physically or chemically attached to surfaces, entrapped in networks or cross-linked in aggregates (CLEA) [9]. Hydrophobic carriers favor the physical adsorption of lipases by means of van der Waals interactions and entropic gain due to water molecule release, whereas H bonding or ion pair formation drives the adsorption onto hydrophilic supports. Although physically immobilized lipases might be partially leached out under industrial conditions their preparation is usually simple, cheap and the support can be reused. Hydrophobic surfaces tend to promote the interfacial activation of lipases, which is crucial for their catalytic efficiency [10,11,12,13,14]. Nevertheless, Ye and coworkers observed that although the adsorption of lipase onto bare polystyrene (PS), a hydrophobic polymer, was more pronounced than onto hydrophilically modified PS, the activity retention of immobilized lipases was higher on the hydrophilic surfaces [15].

Creating novel surfaces which combine chemical functionality and large surface areas, opens the possibility to learn about the behavior of biomolecules immobilized on them. In the present paper spherical nanoparticles with different functionalities were prepared to serve as supports for the immobilization of lipases. PS particles decorated with hydrophilic PEG molecules and hydrophobic Congo red (CR) molecules were synthesized. The catalytic properties of lipases immobilized onto hydrophilic and hydrophobic surfaces were compared with those determined for free lipases. The role played by PEG and Congo red molecules on the catalytic properties of immobilized lipase was discussed. Previous studies showed that PS/PEG particles are adequate supports for the immobilization of bovine serum albumin (BSA), concanavalin A (Con A) or cholesterol oxidase (ChOx) [16,17,18]. However, in the case of immobilized ChOx onto PS/PEG, no enzymatic activity could be observed, probably due to ChOx denaturation, unfavorable orientation or low accessibility of the substrate (cholesterol) to the active site positioned in the hydrated PEG environment [18]. The modification of PS/PEG particles by the adsorption of CR molecules, which have amphiphilic character and shows ability to bind to PEG [19] and polypeptides [20], overcomes the unfavorable situation. In the case of ChOx, bioconjugation effects between CR and ChOx or cholesterol mediated not only the immobilization but also the activation of ChOx immobilized onto PS/PEG/CR particles [18]. In the present work the immobilization of *Candida rugosa* lipases onto hydrophilic PS/PEG or hydrophobic PS/PEG/CR particles was investigated by adsorption isotherms, light scattering and zeta-potential (*ζ*) measurements. Although the adsorption of lipases onto hydrophobic surfaces tends to be more favorable than onto hydrophilic supports, the dependence of activity retention on the surface hydrophobicity seems controversial [15]. *C. rugosa* lipase was chosen because it is one of the most frequently used enzymes in biotechnological processes. It can be produced in large scale with good quality. *C. rugosa* lipase is composed of a mixture of isoenzymes (Lip1–Lip5) with specific catalytic properties, being Lip1 the mostly found isoenzyme in the commercial samples. In spite of the fact that the relationship between the fermentation conditions, isoenzymes structures and biocatalytical performances might be rather complex, *C. rugosa* lipases show large potential for biotechnological applications [19]. The kinetic parameters determined at 25 °C for the hydrolysis of *p*-nitrophenyl butyrate (*p*-NPB) catalyzed by free lipases and for lipases immobilized onto PS/PEG/CR particles were comparable, and they were superior to those determined for lipases immobilized onto PS/PEG particles. Bioconjugation effects between CR and lipases or *p*-NPB were evaluated by means of circular dichroism and spectrophotometry. Thermal stability of immobilized lipases was compared to that of free lipases.

## 2. Results and Discussion

### 2.1. Characteristics of PS/PEG and PS/PEG/CR Particles

**Figure 1:**
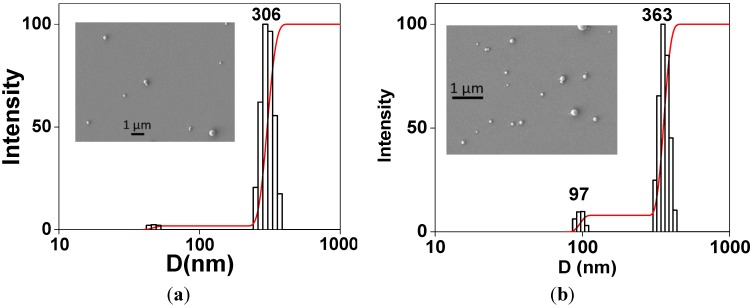
Particle size distribution histograms determined for (**a**) PS/PEG and (**b**) PS/PEG/CR particles. The insets correspond to SEM images taken for dried dispersions.

Dispersions of PS/PEG and PS/PEG/CR presented size distribution histograms, as shown in Figure 1a,b, respectively. Table 1 presents the mean diameter (*D*), polydispersity (*P*) and zeta-potential (*ζ*) values determined from triplicates for PS/PEG and PS/PEG/CR particles. The mean *D* values determined by DLS corroborated with the typical particle sizes observed by scanning electron micrographies (insets in Figure 1a,b). One should note that the preparation of PS/PEG/CR particles corresponds to the adsorption plateau of ~5.0 ± 0.2 µmol∙g^−1^, as described elsewhere [18]. After CR adsorption the average particle size didn’t increase significantly, but the *P* values increased 20%. A small contribution of particles with *D* ~ 100 nm appeared (Figure 1b), which might be due to partial aggregation induced by the CR adsorption.

The *ζ*-potential of (−50 ± 5) mV observed for PS/PEG particles is a consequence of the dissociation of sulfate groups (from the polymerization initiator potassium persulfate) at the interface, which has ionic strength different from that of the bulk due to the electric double-layer. After CR adsorption the *ζ*-potential changed to (−36 ± 5) mV. Interactions between partially protonated CR amine groups and sulfate groups or ethylene glycol oligomers on the PS/PEG particles drive the adsorption of CR onto PS/PEG particles, causing the reduction in the *ζ*-potential values.

**Table 1:** Mean diameter (*D*), polydispersity (*P*) and zeta-potential (*ζ*) values determined for PS/PEG and PS/PEG/CR particles before and after lipase adsorption at ^(a)^ Q_lipase_ = (85 ± 3) nmol∙mg^−1^and ^(b)^ Q_lipase_ = (105 ± 5) nmol∙mg^−1^.

	*D* (nm)	*ζ* (mV)	*P*
PS/PEG	286 ± 15	−(50 ± 5)	0.17 ± 0.01
PS/PEG/CR	290 ± 19	−(36 ± 5)	0.21 ± 0.02
PS/PEG/lipase ^(a)^	380 ± 20	−(32 ± 4)	0.20 ± 0.03
PS/PEG/CR/lipase ^(b)^	405 ± 11	−(25 ± 2)	0.27 ± 0.05

For the sake of comparison the synthesis of PS particles was carried out using K_2_S_2_O_8_ as initiator, but instead of Tween-20 as emulsifier, sodium dodecyl sulfate (SDS) was employed; these particles were coded as PSS. The particles presented mean diameter of (342 ± 20) nm and zeta potential value of (−64 ± 2) mV. The PSS and PS/PEG particles carry sulfate charges on the surface, but only PS/PEG particles are decorated with ethylene glycol oligomers. The adsorption experiments of CR onto PSS particles were performed in the same way as those for CR onto PS/PEG particles. However, the amount of CR adsorbed onto PSS was only 5% of that of CR onto PS/PEG particles. The main reason for this is the electrostatic repulsion between sulfate groups on PSS particles and CR sulfonate groups. These findings show that the interaction between ethylene glycol oligomers on the PS/PEG particles is crucial for the CR adsorption. The use of cationic surfactant, namely, cetyltrimethylammonium bromide (CTAB), for the synthesis of PS particles led to colloidal instability and partial aggregation already before CR adsorption experiments.

### 2.2. Adsorption Behavior of Lipase onto PS/PEG or PS/PEG/CR Particles

**Figure 2:**
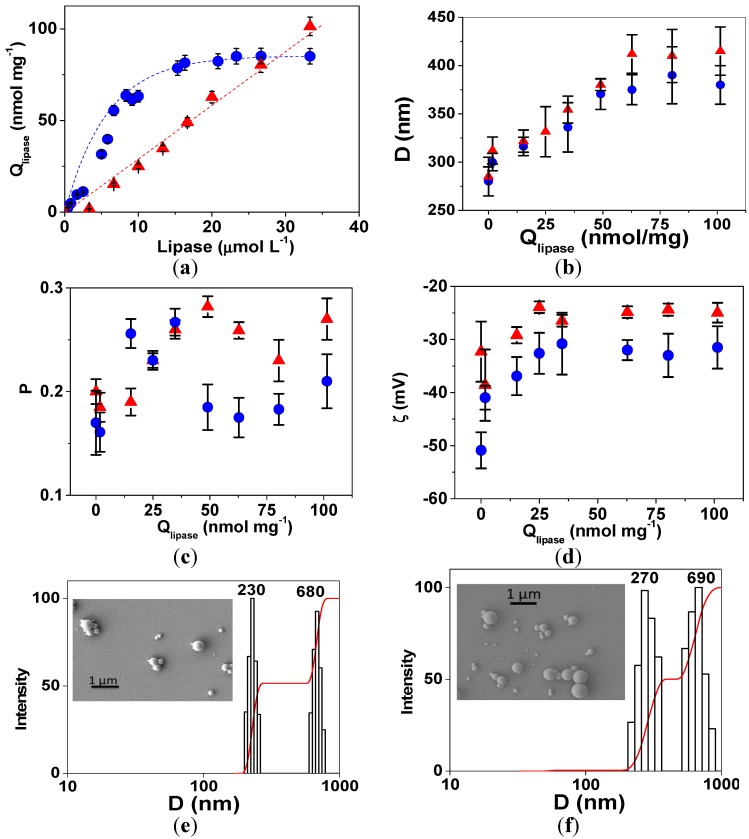
(**a**) Adsorption isotherms determined at (24 ± 1) °C and pH 7.0 for lipase onto PS/PEG (blue circles) and PS/PEG/CR particles (red triangles). The lines are guides for the eyes. (**b**) Mean *D*, (**c**) PD and (**d**) *ζ*-potential values determined for PS/PEG or PS/PEG/CR particles after lipase adsorption as a function of Q_lipase_. Particle size distribution histograms and SEM images (insets) obtained for (**e**) PS/PEG and (**f**) PS/PEG/CR particles after lipase adsorption, Q_lipase_ = 85 ± 3 nmol∙mg^−1^ and Q_lipase_ = 105 ± 5 nmol∙mg^−1^, respectively. Each point is the average of triplicates ± SD.

The adsorption isotherms of lipase onto PS/PEG or PS/PEG/CR particles at (24 ± 1) °C and pH 7 are presented in Figure 2a. The adsorbed amount of lipase (Q_lipase_) onto PS/PEG particles increased linearly with the lipase concentration until the adsorption plateau at (85 ± 3) nmol∙mg^−1^ was achieved. On the other hand, Q_lipase_ onto PS/PEG/CR particles increased linearly and continuously with the lipase concentration. For lipase concentrations smaller than 27 µmol∙L^−1^ the Q_lipase_ values onto PS/PEG were higher than those determined for lipase onto PS/PEG/CR particles. However, for lipase bulk concentrations larger than 27 µmol∙L^−1^ the opposite trend is observed. The largest Q_lipase_ value determined for lipase onto PS/PEG/CR particles was (105 ± 5) nmol∙mg^−1^. The experimental isotherms were not fitted to the theoretical models because in both cases the adsorption is irreversible (less than 10% desorption) and the surfaces are not chemically homogeneous due to the presence of sulfate, ethylene glycol oligomers and CR functional groups.

The mean *D* values determined for PS/PEG or PS/PEG/CR particles after lipase adsorption increased with Q_lipase_ (Figure 2b) up to *D* values of 405 ± 11 nm and 380 ± 20 nm, corresponding to plateau value (Q_lipase_ = 85 ± 3 nmol∙mg^−1^) for PS/PEG particles, and to the highest value (Q_lipase_ = 105 ± 5 nmol∙mg^−1^) for PS/PEG/CR particles, respectively, as presented in Table 1. For both systems the PD values fluctuated from 0.15 to 0.30 (Figure 2c), probably due to the bimodal size distribution evidenced by the histograms in Figure 2e,f, which indicate one population has the typical size of isolated particles and the second population has mean size corresponding to doublets, as evidenced by scanning electron micrographies (insets in Figure 2e,f).

Upon increasing Q_lipase_, the *ζ*-potential values (in modulus) decreased up to constant values of (−32 ± 4) mV and (−25 ± 2) mV, for PS/PEG and PS/PEG/CR, respectively (Figure 2d). The adsorption of lipases on PS/PEG surfaces might be driven by electrostatic interaction between lipase positively charged residues and sulfate groups on the PS/PEG particles. Although at pH 7 the lipase net charge is negative because its isoelectric point (pI) is 4.5 [21], there are patches of positively charged residues that are able to bind to the negatively charged surface, reducing the module of *ζ*-potential values. Similar behavior was observed for cholesterol oxidase onto PS/PEG particles [18] or bovine serum albumin (BSA) onto polyanions [22,23]. In the case of PS/PEG/CR not only the electrostatic interaction but also the hydrophobic interactions drive the immobilization of lipases and reduction of the modulus of *ζ*-potential values. Therefore, the reduction in *ζ*-potential modulus values decreases the electrostatic repulsion among the particles and favors the partial formation of doublets by van der Waals attraction.

One important aspect regards the monomeric or dimeric state of immobilized lipases. This issue was treated in details by the group of Guisan and Fernández-Lafuente [24,25,26,27,28]. At moderate lipase concentrations the dimerization takes place in solution and at the support due to interactions between the hydrophobic areas of the lipase surface that surround the active site of the open form of two lipase molecules. However, in the presence of detergent (0.1% v/v Triton X-100) the dimerization is reduced. The main consequence is that the catalytic properties of lipases immobilized as dimers differ from those of immobilized as monomers [24]. The increase in the mean D values after lipase adsorption on the order of 100 nm shown in Table 1 is mainly due to partial particles aggregation and cannot be discussed in terms of adsorption of lipases monomers or dimers. One strategy to evaluate if lipases were immobilized as monomers or dimers onto PS/PEG or PS/PEG/CR particles is to use flat Si/SiO_2_ or CR covered Si/SiO_2_ wafers to represent the particles and to determine the thickness of adsorbed lipases by means of ellipsometry. Ellipsometry is an optical technique based on the changes of the light polarization state after reflecting from a planar homogeneous isotropic surface, which allows determining the thickness (>0.1 nm) of very thin films, distinguishing monolayers, bilayers or multilayers [29,30]. One should notice that a flat and homogeneous covalently bound PEG layer on a planar surface is not trivially formed. On the other hand, Si/SiO_2_ wafers are very hydrophilic surfaces, as PS/PEG particles, and are convenient surfaces for ellipsometry because they are very flat and homogeneous. Si/SiO_2_ wafers or CR covered Si/SiO_2_ wafers interacted with solutions of lipase at 16.6 μmol L^−1^ (1.0 g/L) and 33 μmol L^−1^ (2.0 g/L) in the absence and in the presence of TritonX-100 at 0.2 mM, under equilibrium conditions at (24 ± 1) °C. The ellipsometric data revealed that regardless the lipase concentration and the presence of Triton X-100, the mean thickness values of adsorbed lipase layer onto Si/SiO_2_ and CR covered Si/SiO_2_ wafers were (1.5 ± 0.2) nm and (1.7 ± 0.2) nm, respectively. Considering the radius of gyration of lipase in water as 1.67 nm [31], the thickness values determined for immobilized lipases onto Si/SiO_2_ and CR covered Si/SiO_2_ wafers were attributed to the formation of lipases monolayer. Thus, if large aggregates were present on the surface, they would yield layers much thicker than those observed for lipases immobilized onto Si/SiO_2_ or CR covered Si/SiO_2_ wafers.

### 2.3. Enzymatic Activity of Free and Adsorbed Lipase

**Figure 3:**
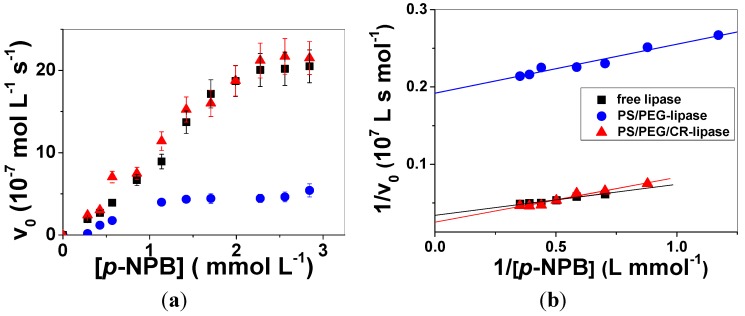
(**a**) Initial rates determined for the hydrolysis of *p*-NPB catalyzed by free lipase (black squares) at 0.4 × 10^−6^ mol/L and lipase immobilized onto PS/PEG (blue circles, Q_lipase_ = 85 ± 3 nmol∙mg^−1^) or onto PS/PEG/CR (red triangles, Q_lipase_ = 105 ± 5 nmol∙mg^−1^) as a function of *p*-NPB concentration. Each point is the average of triplicates ± SD. (**b**) Lineweaver-Burk fitting curves for free lipase (black squares) and lipase immobilized onto PS/PEG (blue circles) and PS/PEG/CR (red triangles).

The enzymatic activity of free and adsorbed lipase onto PS/PEG or PS/PEG/CR particles was monitored by the formation of *p*-NP as a function of time. The dependence of the initial rates of *p*-NP formation on the concentration of *p*-NPB is presented in Figure 3a. For the enzymatic assays with lipase adsorbed onto PS/PEG and PS/PEG/CR particles the highest Q_lipase_ values, namely, Q_lipase_ (85 ± 3) nmol∙mg^−1^ and (105 ± 5) nmol∙mg^−1^, respectively, were employed. For the determination of enzymatic activity of free lipase, the concentration was set at 0.4 µmol∙L^−1^, because it corresponds to the bulk concentration used to achieve the above mentioned Q_lipase_ values (see Experimental Section for details). The experimental data were fitted with Lineweaver-Burk plots, as shown in Figure 3b. The fitting parameters maximum rate (V_max_), Michaelis constant (K_M_), turnover number (*k*_cat_) and catalytic efficiency (ε) are shown in Table 2. The *k*_cat_ values were determined dividing the *V_max_* values by the enzyme bulk concentration (0.4 µmol∙L^−1^). The ε values were calculated dividing *k*_cat_ by K_M_ values.

A comparison among the kinetic parameters determined for the reactions in the presence of free lipases and lipases immobilized onto PS/PEG particles and PS/PEG/CR particles reveals interesting interfacial effects. The smallest K_M_ values were observed for lipases immobilized onto PS/PEG particles, indicating larger affinity for *p*-NPB than free lipase or lipase immobilized onto PS/PEG/CR. The highly hydrated PEG oligomers provided an environment which was more favorable than the others. This finding is in agreement with results reported for the enzymatic activity of lipases immobilized onto hydrophobic and hydrophilic polymeric supports [15]. On the other hand, the V_max_ values determined for lipase onto PS/PEG showed slower enzymatic activity compared to those of free lipases or lipases immobilized onto PS/PEG/CR. This effect is probably due to the hydrophilic nature of PS/PEG particles surfaces, which doesn’t help to promote the lipase open-state conformation. A similar effect was observed for cholesterol oxidases (ChOx), which adsorbed onto PS/PEG particles but presented no enzymatic activity [18]. In solution the effect of PEG on the enzyme conformation and activity seems to depend on the enzyme type and on PEG molecular weight, for instance, PEG 400 changed the conformation of chymotrypsin, retarding the degradation of insulin [32], the enzymatic activity of hexokinase increased 6% in the presence of PEG 400 and decreased in the range 14%–7% in the presence of PEG 1500 and PEG 4000, respectively, due to aggregation [33], PEG 400 or 4000 presented no interaction with glucose-6-phosphate dehydrogenase (G-6-PDH), but favorable interactions were observed between PEG and the co-enzyme NADP^+^, which increased the enzymatic activity in 20% [34]. Comparing the activities in the presence of PEG 200, PEG 8000 or PEG 12000, the enzymatic activity of lipase increased 161% in the presence of PEG 12000 [35]. In the case of PS/PEG particles the PEG chains stem from the surfactant (Tween 20) and have in average only five repeating units, rendering ~200 g/mol. The literature studies cited above indicate that PEG chains are not inert with respect to enzyme conformation; specific interaction and hydration effects might induce changes in chain conformation and orientation.

**Table 2:** Kinetic parameters determined for the hydrolysis of *p*-NPB catalyzed by free lipase at 0.4 × 10^−6^ mol∙L^−1^ and lipase immobilized onto PS/PEG (Q_lipase_ = 85 ± 3 nmol∙mg^−1^) or onto PS/PEG/CR (Q_lipase_ = 105 ± 5 nmol∙mg^−1^). V_max_, K_M_, *k*_cat_ and ε stand for maximal rate, Michaelis constant, turnover number and catalytic efficiency, respectively.

System	V_max_ (10^−7^ mol∙L^−1^∙s^−1^)	K_M_ (10^−3^ mol∙L^−1^)	*k*_cat_ (s^−1^)	ε (L s^−1^∙mol^−1^)
Free lipase	30.3 ± 0.5	1.25 ± 0.08	7.5 ± 0.8	6000 ± 691
PS/PEG/CR-Lipase	46 ± 1	2.9 ± 0.5	11 ± 1	3958 ± 344
PS/PEG-Lipase	5.2 ± 0.5	0.32 ± 0.04	1.3 ± 0.1	4053 ± 678

The K_M_ value found for free lipase was 1.25 ± 0.08 mmol∙L^−1^ (Table 2), similar to the ones determined for chemically immobilized *Candida rugosa* lipase onto amino-terminated polypropylene [36] or for *Rhodotorula* *Glutinis* lipase in the hydrolysis of *p*-NPB [37]. Lipase immobilized onto PS/PEG/CR particles presented K_M_ value of 2.9 ± 0.5 mmol∙L^−1^, indicating that the affinity for *p*-NPB decreased in comparison to that in solution or onto PS/PEG particles. However, the largest V_max_ and *k*_cat_ values were achieved for lipases adsorbed onto PS/PEG/CR particles. These findings indicate that although the affinity between lipase and *p*-NPB was not the largest among the three systems, the presence of CR molecules on the polymer particles turned the environment more hydrophobic, which is suitable for the interfacial activation of lipases [38]. The latter requires: (i) lid opening with consequent conformational changes and (ii) orientation of substrate at the interface. The amphiphilic character of CR molecules due to the hydrophobic aromatic rings and amine and sulphonate polar groups seems to favor the interaction between lipase active site and *p*-NPB, keeping the hydration at a reasonable level. The enzymatic activity of physically bound lipases tend to be similar or even larger than that of free lipase, when the supports are partially hydrophobic but carry hydrophilic groups that keep the hydration level necessary to the hydrolysis reactions. For instance, lipase immobilized onto cellulose ester films exhibited activity higher than that of free lipase and could be recycled three times and stored for one month keeping the activity at a reasonable level [10]. The interaction between the hydrophobic moieties of cellulose esters and the lid helped to keep the open-state conformation, while the hydroxyl groups of glycosidic rings interacted with water molecules. Other supports with similar behavior are zirconia nanoparticles modified by surfactant molecules [11], cyclodextrin-based carbonate nanosponge [12], ethylene–vinyl alcohol polymer functionalized with acyl chlorides [13], multiwalled carbon nanotubes modified by amino-cyclodextrin [14].

**Figure 4:**
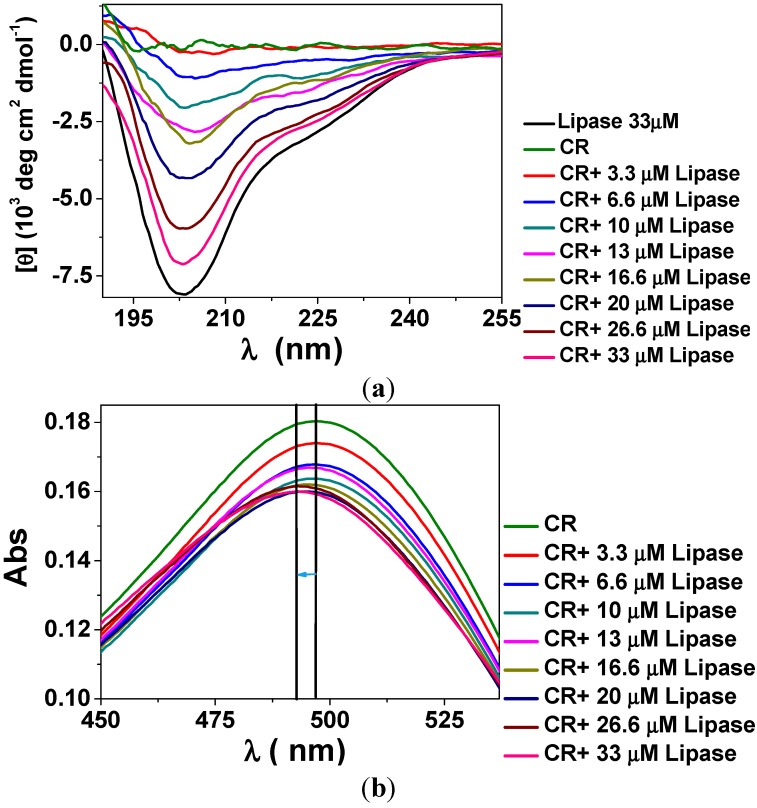
(**a**) Circular dichroism (CD) and (**b**) electronic spectra obtained for mixtures of CR at 8.0 μmol∙L^−1^ and lipase at increasing concentration from 3.3 µmol∙L^−1^ to 33 µmol∙L^−1^.

In order to gain insight about the interactions between CR molecules and lipase, circular dichroism (CD) and electronic spectra were taken for mixtures of CR at 8.0 μmol∙L^−1^ and lipase at increasing concentration from 3 µmol∙L^−1^ to 33 µmol∙L^−1^, as shown in Figure 4a,b, respectively. CR presented no CD signal in the range between 190 nm and 260 nm. CD spectrum obtained for pure lipase corresponds to ~22% of helical structure, which is typical for lipases [39], with minimum at ~204 nm and broad shoulder in the range of 220 nm to 225 nm (Figure 4a). Such typical features could be observed in the mixtures of lipase at concentrations larger than 13 µmol∙L^−1^ and CR, indicating that CR molecules do not promote lipase conformational changes. For lipase concentration smaller than 13 µmol∙L^−1^ the CD signal became too weak.

The electronic spectra obtained for pure CR at 8.0 μmol∙L^−1^ (Figure 4b) presented two π → π* transitions, one at 497 nm (the strongest) and another at 350 nm (not shown), and low-intensity n → π* transition associated to the azo group [40]. As lipase was added to the system a blue shift (hypsochromic effect) in the maximum was detected. At the highest lipase concentration (33 µmol∙L^−1^) the shift was 5 nm. Such hypsochromic effect is generally due to increase of medium polarity and has been already observed for mixtures of CR and cholesterol oxidase [18]. Thus, the blue shift observed in Figure 4b might indicate bioconjugation mediated by H bonding between CR amine groups and lipase polar residues. Electronic spectra obtained for mixtures of CR at 8.0 μmol∙L^−1^ and *p*-NPB in the concentration range between 0.3 mmol∙L^−1^ and 3.0 mmol∙L^−1^ showed no shift regarding the absorption band at 497 nm, indicating no bioconjugation effect between CR and *p*-NPB.

**Figure 5:**
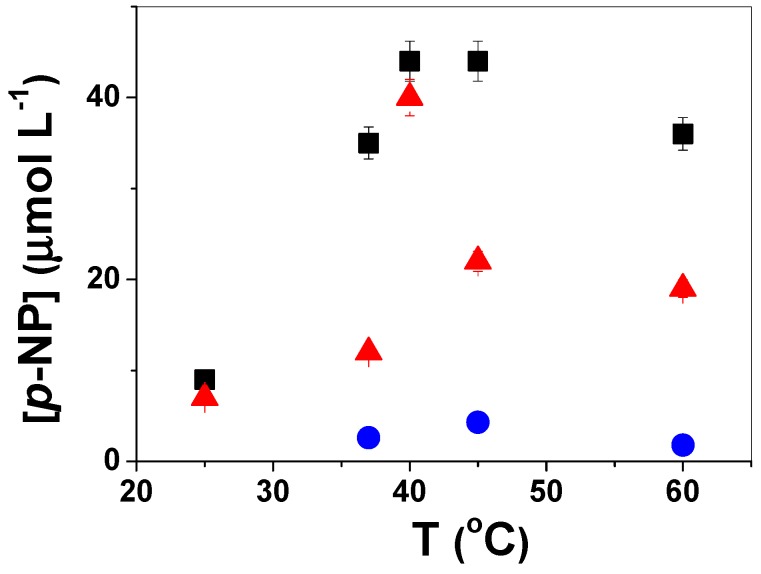
Concentration of *p*-NP formed from the hydrolysis of *p*-NPB catalyzed by free lipases (black squares), lipases immobilized onto PS/PEG/CR (red triangles, Q_lipase_ = 105 ± 5 nmol∙mg^−1^) and PS/PEG (blue circles, Q_lipase_ = 85 ± 3 nmol∙mg^−1^) particles at 25 °C, 37 °C, 40 °C, 45 °C and 60 °C. Each point is the average of triplicates ± SD.

The thermal stability of free and immobilized lipases was investigated by monitoring the formation of *p*-NP after 20 min hydrolysis in the temperature range from 25 °C to 60 °C, as shown in Figure 5. No shift of optimal temperature was observed for lipases immobilized onto PS/PEG/CR particles (red triangles) in comparison to free lipases (black squares), since for both systems the highest hydrolytic activity was observed at 40 °C. At 60 °C free lipase and lipase immobilized onto PS/PEG/CR presented ~80% and ~50% of the activity measured at 40 °C. Lipases immobilized onto chitosan presented similar behavior [41]. Regardless the temperature, the activity observed for lipases immobilized onto PS/PEG particles was very low in comparison to those determined for free lipase or lipases immobilized onto PS/PEG/CR particles.

**Figure 6:**
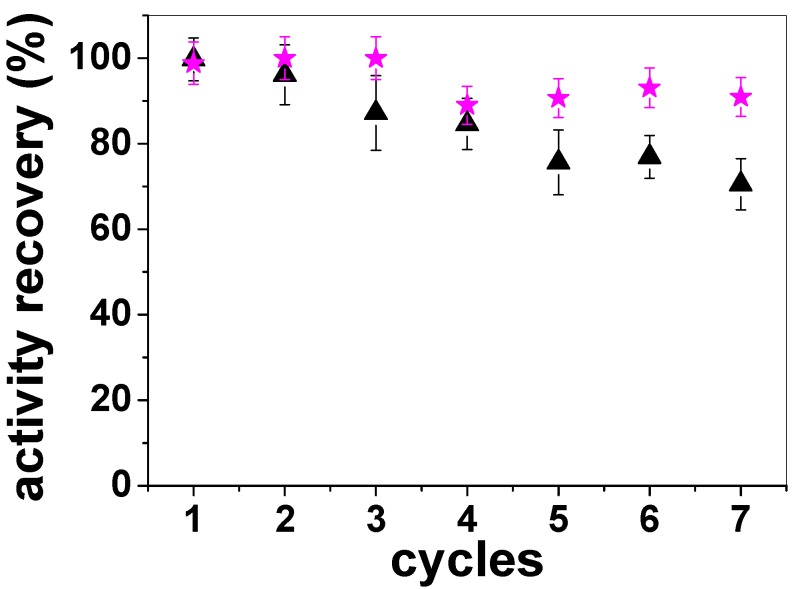
Activity recovery determined at 25 °C and 40 °C for lipases immobilized onto PS/PEG/CR particles (Q_lipase_ = 105 ± 5 nmol∙mg^−1^). Each point is the average of triplicates ± SD.

Lipase immobilized onto PS/PEG/CR particles (Q_lipase_ = 105 ± 5 nmol∙mg^−1^) could be recycled consecutively seven times. Figure 6 presents the activity recovery (%), which is the activity of immobilized lipase compared to that of total starting activity of free lipase [9], determined at 25 °C (black triangle) and 40 °C (pink star). The enzymatic activities of free and immobilized lipase were comparable in the first use at both temperatures. Then the activity recovery decreased with the number of consecutive reactions, so that at the seventh hydrolysis reaction the activity recovery was approximately 70% and 90% at 25 °C and 40 °C, respectively. These findings revealed that the stability of lipases immobilized onto PS/PEG/CR is favored at 40 °C and deactivations by desorption or conformational changes are minimal. Thus, lipases immobilized onto PS/PEG/CR particles are advantageous because the cumulative activity after seven reactions overcomes in approximately 6-fold the activity of free lipase, which cannot be recovered for reuse. Moreover, lipases immobilized onto PS/PEG/CR particles can be stored for 7 days at room temperature, keeping the original catalytic activity. On the other hand, lipases immobilized onto PS/PEG particles (Q_lipase_ = 85 ± 3 nmol∙mg^−1^) could not be recycled, although the K_M_ values for this system was the smallest one (Table 2).

## 3. Experimental Section

### 3.1. Materials

**Scheme 1:**
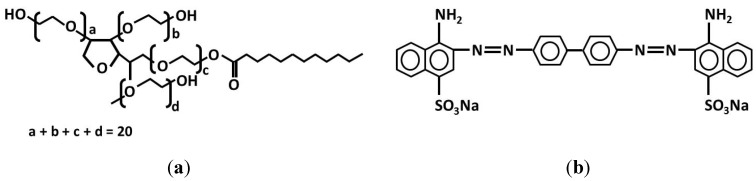
Representation of chemical structures of (**a**) Tween-20 and (**b**) Congo red (CR).

Styrene (S; Fluka, St. Gallen, Switzerland), potassium persulfate (K_2_S_2_O_8_, Merck, Darmstadt, Germany) and poly(ethylene glycol) sorbitan monolaurate (Tween-20, Sigma-Aldrich Co., St. Louis, MO, USA) were used in the emulsion polymerization. Congo red (CR) was provided by Sigma-Aldrich (São Paulo, Brazil). Chemical structures of Tween-20 and CR are schematically shown in the Scheme 1. Lipase from *Candida rugosa* (~60 kDa [42], isoelectric point at pH 4.5 [21], L1754) and *p*-nitrophenyl butyrate, (*p*-NPB, N9876) were provided by Sigma-Aldrich (Brazil). All reagents were used as received. Silicon (Si/SiO_2_) wafers (100) with a native silicon oxide layer of approximately 2 nm thick from University Wafers (Boston, MA, USA), cut in a typical dimension of 1 cm^2^ and rinsed in a standard manner [10,30] were used as substrates for ellipsometry.

### 3.2. Methods

#### 3.2.1. Preparation of PS/PEG and PS/PEG/CR Particles

The PS/PEG particles were synthesized following a typical emulsion polymerization recipe, as described elsewhere [16,17,18]. The polymerization took place in a four neck flask containing 100 mL of Tween-20 solution at 4.8 × 10^−5^ mol∙L^−1^, which is close to the critical micelle concentration (c.m.c.). The system was purged with N_2_ during the whole process and the polymerization was carried out under reflux with water and mechanical stirring at 500 rpm. The temperature was set to (75 ± 2) °C, then, 10 mL of styrene were added and the temperature was brought up to (80 ± 2) °C. Afterwards, 1.0 g of K_2_S_2_O_8_ (initiator) was added. The synthesis was carried out for 2 h. Then, the system was cooled to room temperature. The dispersion was dialyzed (dialysis membrane 14,000 MW; Viskase Corp., Darien, IL, USA) until ionic conductivity achieved 5 μS∙cm^−1^.

From a previous study [18] one observed that the adsorbed amount of CR (Q_CR_) onto polymer particles at (24 ± 1) °C and pH 7 increased with CR concentration up to the onset for the adsorption plateau, at 7.3 µmol∙L^−1^ CR, and Q_CR_ value of 4.73 ± 0.02 µmol∙g^−1^. Therefore, we used this experimental condition to cover the PS/PEG particles with CR molecules.

#### 3.2.2. Adsorption Isotherms of Lipase onto PS/PEG or PS/PEG/CR Particles

The adsorption isotherms for lipase onto PS/PEG or PS/PEG/CR particles were obtained at (24 ± 1) °C and pH 7, in the lipase concentration range of 3 µmol∙L^−1^ to 33 µmol∙L^−1^ and constant N_P_ = 1.7 × 10^10^ particles mL^−1^. Lipase and nanoparticles interacted for 24 h under constant rotation at 3 rpm in a home-made vertical carrousel. The adsorption time of 24 h was enough to assure equilibrium conditions. Then the dispersions were centrifuged at 12,045 g for 30 min, and their supernatants were separated from the particles. The concentration of free lipase in the supernatants was determined by UV-Vis spectrophotometry at 280 nm in a DU640 spectrophotometer (Beckman-Coulter, Brea, CA, USA) using molar absorptivity ε = 76365 (L∙mol^−1^∙cm^−1^) [10]. The medium pH and temperature used for the calibration curve were the same as those used in the adsorption experiments. The concentration of adsorbed lipase onto the particles was calculated as the difference between the initial lipase concentration and the free lipase concentration in the supernatant. The adsorbed amount of lipase (Q_lipase_) onto polymer particles was calculated dividing the concentration of adsorbed lipase by the mass of PS/PEG or PS/PEG/CR particles in the dispersion at N_P_ = 1.7 × 10^10^ particles mL^−1^. Thus the unit corresponding to Q_lipase_ is µmol∙g^−1^.The particle mean diameter (*D*), polydispersity (P) and mean *ζ*-potential were determined after each adsorption experiment, centrifugation, complete supernatant removal for the quantification of free lipase and re-dispersion in KCl 10 mmol∙L^−1^, see details below.

#### 3.2.3. Characterization of PS/PEG and PS/PEG/CR Particles before and after Lipase Adsorption

Particle size (zeta-average diameter, *D*), size distribution, polydispersity (*P*) and zeta-potential (*ζ*) values were determined with the ZetaPlus-ZetaPotential Analyzer (Brookhaven Instruments Corporation, Holtsville, NY, USA), which was equipped with a 677 nm laser and dynamic light-scattering (DLS) photon correlation spectroscopy at 90° for particle sizing. Mean diameter values were obtained by fitting data to log-normal size distributions which do not discriminate between one, two, or more different populations and considers always all scattering particles as belonging to one single Gaussian population. On the other hand, for the size distribution and *P* data, fitting was performed by the apparatus software using the non-negatively constrained least squares algorithm, which is a model independent technique allowing to achieve multimodal distributions [43]. Zeta-potential (*ζ*) was determined from electrophoretic mobility (µ) in 10 mmol∙L^−1^ KCl and the Smoluchowski equation *ζ* = µη/ε, where η is the medium viscosity and ε the medium dielectric constant.

The mean particle number density (N_P_) was calculated considering the particle mean diameter calculated by DLS and the solid content, as described elsewhere [44].

In order to gain more insight about the adsorption behavior of lipase onto PS/PEG or PS/PEG/CR particles, the particles were characterized before and after lipase adsorption with Np on the order of 10^8^ particles mL^−1^. After lipase adsorption the dispersions were centrifuged, the supernatant was replaced by pure water and the particles were re-dispersed. This procedure was repeated once more and then the size distribution and zeta potential values were analyzed.

Scanning electron microscopy (SEM) analyses were performed to determine the morphology of dried particles using SEM-FEG JEOL 7401F equipment (Jeol, Tokyo, Japan). The stock dispersions were diluted 1,000 times in Milli-Q water; then droplets were deposited onto clean Si wafers. The water evaporated slowly at room temperature. The dried dispersions were coated with a 3 nm gold layer.

#### 3.2.4. Desorption Experiments

Lipase covered PS/PEG or PS/PEG/CR particles were submitted to desorption tests. After lipase adsorption and centrifugation the supernatant was removed from the tubes and initial volume was completed with pure solvent (distilled water). The dispersions were kept for 24 h under constant rotation at 3 rpm. The dispersions were then centrifuged at 12,045 g for 30 min and the absorbance of the supernatant was measured in order to determine the concentration of desorbed lipase. The desorbed amount didn’t change significantly after 24 h in contact with pure solvent.

#### 3.2.5. Catalytic Activity of Immobilized Lipase and Free Lipase

**Scheme 2:**
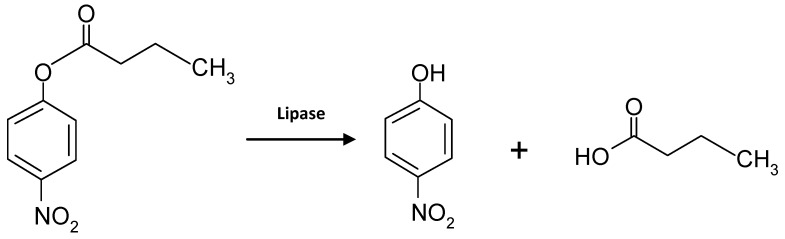
Representation of *p*-NPB hydrolysis catalyzed by lipase, yielding butyric acid and (*p*-NP).

The activity of free lipase and lipase immobilized onto PS/PEG or PS/PEG/CR particles was determined by measuring the formation of *para*-nitrophenol (*p*-NP), as a result of enzymatic hydrolysis of (*p*-NPB), as indicated in Scheme 2.

The formation of *p-*NP was monitored by means of a Beckmann Coulter DU 640 UV-VIS spectrophotometer operating at a fixed wavelength of 400 nm and (24 ± 1) °C. The concentration of *p-*NP formed was calculated using molar extinction coefficient ε = 13800 L mol^−1^ cm^−1^ [45]. First, 25 μL of *p*-NPB (M 209.2 g/mol, density 1.19 g/cm³) was dissolved in 5.0 mL of isopropanol. After that, *p-*NPB solution was added to 45 mL of Trisma-HCl 0.050 mol/L buffer (pH 7.5), yielding the concentration of stock solution of *p*-NPB as 2.8 × 10^−3^ mol/L. The kinetic parameters were determined in the *p*-NPB concentration range from 0.3 × 10^−3^ mol/L to 2.8 × 10^−3^ mol/L at 25 °C. The volume and concentration of each reagent mixed in the spectrophotometric cell were the following: 20 µL of dispersions containing lipase immobilized onto PS/PEG (Q_lipase_ = 85 ± 3 nmol∙mg^−1^) or PS/PEG/CR (Q_lipase_ = 105 ± 5 nmol∙mg^−1^), a given volume of stock solution of *p*-NPB to achieve the desired concentration and 50 mM Tris-HCl buffer to complete the total volume of 1.0 mL. In order to compare the catalytic efficiency between free and immobilized lipase, the concentration of free lipase was set as 18 µmol∙L^−1^ because it corresponds to the highest Q_lipase_ onto PS/PEG or PS/PEG/CR particles. Thus, the final concentration of free lipase or lipase immobilized onto polymeric particles in the spectrophotometric cell was 0.4 μmol∙L^−1^. Control experiments were performed, where bare PS/PEG or bare PS/PEG/CR particles were added to the enzymatic reaction in the absence of lipase and the formation of *p*-NP was monitored by UV-Vis spectrophotometry (λ_max_ = 400 nm) as a function of time. In a similar way, the activity of lipase immobilized onto PS/PEG or PS/PEG/CR particles was determined at 25 °C and 40 °C after seven consecutive recycles or after storing during seven days in the laboratory environment.

Thermal stability of free and immobilized lipases was investigated by monitoring the concentration of *p*-NP formed during 20 min in the temperature range between 25 °C and 60 °C. The volume and concentration of each reagent mixed in the spectrophotometric cell were the following: 20 µL of dispersions containing lipase immobilized onto PS/PEG (Q_lipase_ = 85 ± 3 nmol∙mg^−1^) or PS/PEG/CR (Q_lipase_ = 105 ± 5 nmol∙mg^−1^), 250 µL of stock solution of *p*-NPB to achieve 0.7 × 10^−3^ mol∙L^−1^ and 50 mM Tris-HCl buffer to complete the total volume of 1.0 mL.

#### 3.2.6. Evaluation of Bioconjugation Effects

Circular dichroism (CD) measurements were performed on a Jasco J-720 spectropolarimeter (Jasco, Easton, MD, USA), in the wavelength range of 190 nm to 260 nm, at (24 ± 1) °C, using a 0.5 cm quartz cell. All CD spectra were collected at pH 7 using scan speed of 50 nm∙min^−1^, time constant of 0.5 s and bandwidth of 1 nm. Eight scans were accumulated for each sample. The samples for CD were prepared with lipase solutions at concentration range of 3 µmol∙L^−1^ to 33 µmol∙L^−1^ in the absence and in the presence of CR at constant concentration of 8.0 μmol∙L^−1^. More diluted lipase solutions yielded no CD signal.

Changes in the electronic spectra of CR in the visible range were monitored by increasing addition of lipase. The CR concentration was kept constant at 8.0 µmol∙L^−1^, while the lipase concentration increased from of 3 µmol∙L^−1^ to 33 µmol∙L^−1^. Bioconjugation between CR molecules and *p*-NPB was evaluated by monitoring the changes in the electronic spectra of CR at 8.0 µmol∙L^−1^ in the presence of *p*-NPB in the concentration range of 0.3 × 10^−3^ mol∙L^−1^ to 2.8 × 10^−3^ mol∙L^−1^.

#### 3.2.7. Ellipsometry

The mean thickness of the adsorbed lipase layers was determined by ellipsometry using a vertical computer-controlled DRE-EL02 ellipsometer (Ratzeburg, Germany) with an angle of incidence ϕ of 70.0° and wavelength λ of 632.8 nm He−Ne laser. Using the fundamental ellipsometric equation [29]:

e^iΔ^ tan Ψ = (*R_p_/R_s_*) = *f* (n_k_, d_k_, λ, φ) (1)

where *R_p_* and *R_s_* are the overall reflection coefficients for parallel and perpendicular waves, respectively. The mean thickness d_k_ and refractive index n_k_ can be calculated from variation of the ellipsometric angles Δ and Ψ using iterative calculations and a multilayer model composed by the substrate, the unknown layer, and surrounding medium. The thickness of the SiO_2_ layer was determined in air, considering the indices of refraction for Si as n_Si_ = (3.88 − i0.018), n_air_ = 1.00 and n_SiO2_ = 1.462 and “infinite” thickness for the Si and air layers. The mean thickness obtained for SiO_2_ was (2.0 ± 0.1) nm. For the CR adsorbed layer onto Si/SiO_2_, n_CR_ was 1.59 and d_CR_ = (1.4 ± 0.2) nm. After that the thickness values for the lipase layer, d_lipase_, adsorbed onto Si/SiO_2_ or Si/SiO_2_/CR were calculated using refractive index n = 1.52 [10]. The d_lipase_ values were calculated in the air just after the equilibrium adsorption.

## 4. Conclusions

Lipases adsorbed physically onto polymeric nanoparticles decorated either with PEG or with CR. The affinity of *p*-NPB for lipases immobilized onto PS/PEG particles was larger than that for free lipases or for lipases immobilized onto PS/PEG/CR particles. On the other hand, the hydrolysis rate observed for lipases immobilized onto PS/PEG/PS particles was larger than those observed for free lipases or for lipases onto PS/PEG particles. The main reasons for this are bioconjugation effects, which favor: (i) the interactions between the hydrophobic moieties of CR and the lid, maintaining the open state conformation and (ii) hydrated environment close to the active site to enable the hydrolysis reaction. Thus, although the affinity between lipase immobilized onto PS/PEG and the substrate was high, the open state conformation was unfavored. PS/PEG/CR particles are easily prepared and cheap (~US$ 1.00 per liter of dispersions with N_P_ = 1.7 × 10^14^ particles L^−1^). For practical purposes, the immobilization of lipases on them is advantageous because they can be used efficiently as catalysts, retaining their catalytic properties after seven repeated cycles, and can be stored in the laboratory environment over seven days retaining activity at a high level.
